# BACL Is a Novel Brain-Associated, Non-NKC-Encoded Mammalian C-Type Lectin-Like Receptor of the CLEC2 Family

**DOI:** 10.1371/journal.pone.0065345

**Published:** 2013-06-11

**Authors:** Olga Lysenko, Dorothea Schulte, Michel Mittelbronn, Alexander Steinle

**Affiliations:** 1 Institute for Molecular Medicine, Goethe-University Frankfurt am Main, Frankfurt am Main, Germany; 2 Institute of Neurology (Edinger Institute), Goethe-University Frankfurt am Main, Frankfurt am Main, Germany; Hannover Medical University (MHH), Germany

## Abstract

Natural Killer Gene Complex (NKC)–encoded C-type lectin-like receptors (CTLRs) are expressed on various immune cells including T cells, NK cells and myeloid cells and thereby contribute to the orchestration of cellular immune responses. Some NKC-encoded CTLRs are grouped into the C-type lectin family 2 (CLEC2 family) and interact with genetically linked CTLRs of the NKRP1 family. While many CLEC2 family members are expressed by hematopoietic cells (e.g. CD69 (*CLEC2C)*), others such as the keratinocyte-associated KACL (*CLEC2A*) are specifically expressed by other tissues. Here we provide the first characterization of the orphan gene *CLEC2L*. In contrast to other CLEC2 family members, *CLEC2L* is conserved among mammals and located outside of the NKC. We show that *CLEC2L*-encoded CTLRs are expressed as non-glycosylated, disulfide-linked homodimers at the cell surface. *CLEC2L* expression is fairly tissue-restricted with a predominant expression in the brain. Thus *CLEC2L*-encoded CTLRs were designated BACL (brain-associated C-type lectin). Combining *in situ* hybridization and immunohistochemistry, we show that BACL is expressed by neurons in the CNS, with a pronounced expression by Purkinje cells. Notably, the *CLEC2L* locus is adjacent to another orphan CTLR gene (*KLRG2*), but reporter cell assays did neither indicate interaction of BACL with the KLRG2 ectodomain nor with human NK cell lines or lymphocytes. Along these lines, growth of BACL-expressing tumor cell lines in immunocompetent mice did not provide evidence for an immune-related function of BACL. Altogether, the *CLEC2L* gene encodes a homodimeric cell surface CTLR that stands out among CLEC2 family members by its conservation in mammals, its biochemical properties and the predominant expression in the brain. Future studies will have to reveal insights into the functional relevance of BACL in the context of its neuronal expression.

## Introduction

The mammalian Natural Killer Gene Complex (NKC) represents a cluster of genes encoding for C-type lectin-like receptors (CTLRs) mainly expressed on hematopoietic cells such as myeloid cells, T cells or the name giving Natural Killer (NK) cells [1], [2]. Common hallmark of NKC-encoded CTLRs is a type II transmembrane topology with a single extracellular C-type lectin-like domain (CTLD) engaged in binding of proteinaceous ligands instead of carbohydrates, the typical ligands of lectins [2]. The CTLD is characterized by six conserved cysteins and a hydrophobic ‘WIGL’ motif that stabilize the lectin-like fold by forming intramolecular disulfide bonds and a hydrophobic core, respectively [2], [3]. NKC-encoded CTLRs are subdivided into ‘killer cell lectin-like receptors’ (KLRs), such as NKG2D, and other ‘C-type lectin molecules’ (CLECs). The latter include members of the CLEC2 family of CTLR that share distinct sequence characteristics [4]. The CLEC2 family comprises CD69, the mouse Clr molecules and the human members LLT1, AICL and KACL, with CD69 being the only NKC-encoded CLEC2 family member conserved in both species [2], [4]. While NKC-encoded KLRs, including members of the NKRP1 subfamily, are expressed on NK cells or other effector lymphocytes [4]–[8], the tissue expression of CLEC2 family members broadly varies. For instance, human molecules LLT1, AICL and KACL were described to be expressed on B-cells, monocytes and keratinocytes, respectively [9]–[11], while transcripts of mouse Clr-d and Clr-f recently were specifically associated with the eye and the intestine, respectively [12]. It has been shown that several CTLRs of the NKRP1 and CLEC2 families that are encoded in the NKC in tight genetic linkage constitute receptor-ligand pairs with certain NKRP1 receptors engaging one or several CLEC2 family members [13]. For example, mouse Nkrp1d was shown to bind Clr-b, while Nkrp1f was shown to bind Clr-c, -d, and -g [13]–[16]. Similarly, human LLT1 engages NKRP1A (CD161) [17], [18] and the related NKp80 and NKp65 receptors bind the adjacently encoded AICL and KACL molecules, respectively [9], [10]. While some of these receptor-ligand pairs inhibit NK cell effector functions (e.g. NKRP1A/LLT1), others (e.g. NKp80/AICL; NKp65/KACL) stimulate NK cell cytotoxicity [9]–[11], [18]. Collectively, there has been quite some progress in defining NKRP1 receptors and their CLEC2 family ligands, paired with insights into their expression, however, the *in vivo* function of these genetically linked receptor/ligand pairs remains poorly understood.

In the present study we investigate the expression of the hitherto uncharacterized CLEC2 family gene *CLEC2L*. We show that *CLEC2L* encodes for a homodimeric cell surface CTLR that is predominantly expressed in the brain and hence was termed BACL (brain-associated C-type lectin).

## Materials and Methods

### Ethics Statement

Buffy coats of healthy volunteers were obtained after written informed consent from the German Red Cross, Frankfurt am Main (approved by the local ethics committee of the University Hospital Frankfurt am Main). Usage of pseudonymized autopsy material (human brain) for research purposes was approved by the local ethics committee of the University Hospital Frankfurt am Main. The need for informed consent has been waived by the Ethical Committee. Animal experiments were approved by the local authorities (Regierungspräsidium Darmstadt, Germany; permit numbers F146/02 and F94/Anz03) and performed in full compliance with the respective national guidelines.

### Cells and Transfectants

Cell lines were purchased from the Deutsche Sammlung von Mikroorganismen und Zellkulturen (DSMZ, Braunschweig, Germany). BWZ.36 reporter cells were a kind gift of N. Shastri, University of California, Berkeley [19]. Peripheral blood mononuclear cells (PBMC) of healthy donors were isolated from buffy coats (German Red Cross, Frankfurt am Main) by density gradient centrifugation. Adherent cells were cultured in DMEM (PAA, Pasching, Austria), suspension cells in RPMI 1640 (Sigma, Steinheim, Germany). All media contained 10% FCS, 2 mM Glutamine, 100 U/ml penicillin and 100 µg/ml streptomycin (all from PAA). For analyses of protein expression, RSV-BACL constructs were transiently transfected into 293T cells using AppliFect reagent (AppliChem, Darmstadt, Germany) according to the manufacturer's instructions. Upon transfection, cells were cultured in complete DMEM medium for 48 h before analysing BACL expression by flow cytometry or immunoblotting. For the generation of stable transfectants, 293 cells were transfected with AppliFect and selected in complete DMEM medium with 1.8 mg/ml G418 sulphate. Transfection of suspension cells (BWZ.36, RMA) was performed by electroporation using 20 µg of linearized plasmid DNA and the Gene Pulser Xcell™ (Bio-Rad, Munich, Germany) (250 V, 950 µF) followed by selection in RPMI 1640 medium containing the appropriate concentration of G418 sulphate (1.8 mg/ml for BWZ.36, 1 mg/ml for RMA).

### Reverse Transcription and Quantitative PCR

Total RNA of most human tissue samples was purchased from Life Technologies (Darmstadt, Germany) except for bone marrow (Clontech, Saint-Germain-en-Laye, France). RNA from PBMC and mouse tissues was isolated using peqGOLD TriFast (Peqlab, Erlangen, Germany). Total RNA from PBMC and mouse organs was treated with DNAse I and reverse transcribed using MMLV-RT RNAse H^-^ mutant and random primers (all from Promega, Mannheim, Germany). Resulting cDNA was subjected to TaqMan® quantitative PCR for 45 cycles on the Applied Biosystems® StepOnePlus™ cycler (Life Technologies, Darmstadt, Germany) with the FAM-labeled probe 5′ CCA GGC TG 3′ (#64 UPL library; Roche, Mannheim, Germany) and the following pairs of oligonucleotides: 5′ GAC CCG TTT GAT CCG GAC A 3′ and 5′ AGT ATA GGC CAT CTT GCT GCA 3′ (hBACL), 5′ ACC CAG ACA CAT TCA CTA TCT C 3′ and 5′ TCA CGT ATA GGC CAT CTT GC 3′ (mBACL). For normalization, amplification of human TBP or eukaryotic 18S rRNA was monitored using TaqMan® endogenous control assays (Life Technologies).

### *In situ* Hybridization

For *in situ* hybridization BACL cDNA was cloned into the pBluescript II KS(+) vector and used as template for *in vitro* transcription with T3 or T7 RNA polymerases and the DIG RNA labeling mix (all from Roche) to generate digoxigenin (DIG)-labeled probes. Subsequently, free nucleotides were removed by RNA precipitation. *In situ* hybridization on vibratome sections was performed as described previously [20]. Briefly, brain tissues from paraformaldehyde (PFA)-perfused adult C57BL/6 mice and human PFA-stored brain tissues were washed with phosphate-buffered saline (PBS), embedded in a BSA-gelatine mix (15% (w/v) BSA, 0.5% (w/v) gelatine in PBS, 2.5% Glutaraldehyde) and cut into sagittal sections (thickness: 80 µm) using a vibratome (Leica, Wetzlar, Germany). Sections were dehydrated by subsequent incubations in increasing concentrations of methanol and stored at −20°C. Prior to hybridization sections were rehydrated, bleached with 6% H_2_O_2_ and equilibrated in a pre-hybridization solution (50% (v/v) formamide, 2% (w/v) blocking reagent (Roche), 0.2 M NaCl, 1.1 mM Tris, 8.9 mM Tris-HCl, 5 mM Na_2_HPO_4_, 5.6 mM NaH_2_PO_4_^.^H_2_O, 5 mM EDTA) at 65°C for 5 h. Subsequently, sections were incubated in hybridization solution (pre-hybridization solution with additional 10% (w/v) dextran sulfate, 1x Denhardt's and 1 mg/ml yeast RNA) containing 1 µg/ml DIG-labeled probe overnight at 65°C. Sections were repeatedly washed with a low stringency buffer (50% formamide/1X SSC) and a high stringency buffer (50% formamide/0.2X SSC) at 65°C. Then sections were blocked with 10% sheep serum (Sigma) in Tris-buffered saline/0.1% Tween-20 (TBST) for 5 h at 4°C and incubated with alkaline phosphatase-conjugated anti-DIG-Fab (Roche) (1∶2000) in 1% sheep serum in TBST overnight at 4°C. Sections were washed 4x 1 h in TBST and developed by addition of nitro blue tetrazolium chloride/5-Bromo-4-chloro-3-indolyl phosphate mix (NBT/BCIP mix, Roche) for at least 20 min. Stained sections were post-fixed with 4% PFA/0.1% Glutaraldehyde for 1 h and mounted onto glass slides for visualization.

### cDNA Cloning, Chimeric Reporter Constructs and Mutagenesis

Total cDNA from brain tissue of a C57BL/6 mouse was used for amplification of the BACL cDNA with the following oligonucleotides: 5′ ATG GAG CCG GCC CGG GAG CC 3′ and 5′ TCA CGT ATA GGC CAT CTT GCT GCA CAC 3′. In a second PCR, NheI and XhoI restriction sites were added for cloning into the RSV.5neo expression vector containing C-terminal FLAG- and hexahistidine tags. Mutagenesis was performed using the QuickChange site-directed mutagenesis kit (Stratagene, Santa Clara, USA) and the RSV-BACL vector. For the generation of reporter constructs the nucleotide sequences for the C-type lectin-like ectodomains were amplified with the following pairs of oligonucleotides: 5′ AGC AAC ATG TGC CCG GAG GAC TG 3′ and 5′ GCT ACT CGA GAG TAT AGG CCA TC 3′ for BACL from human brain cDNA and 5′ AGC AAC ATG TGC CCC CCA GGC TG 3′ and 5′ GCT ACT CGA GCT GGG TCC CCT TG 3′ for KLRG2 from human thyroid gland cDNA. These oligonucleotides contained overlapping sequences for fusion with sequences encoding for the stalk region and transmembrane domain of Ly49A and the cytoplasmic domain of mouse CD3ζ, amplified from an existing reporter construct (NKG2D-Ly49A-CD3ζ). The resulting fusion products were equipped with NheI and XhoI restriction sites and cloned into the RSV.5neo expression vector containing C-terminal FLAG- and hexahistidine tags. All cloned PCR products were verified by restriction digestion and sequencing.

### Generation of Soluble BACL-ectodomains and Antibody Production

The ectodomain of BACL was cloned into the pSec-Tag2 vector by restriction free cloning. To this aim, a PCR with the oligonucleotides 5′ GAA TGG CAC GAA AAG CCG GCC TCC AAG GGC TGC ATC AAG TG 3′ and 5′ GAT CCT CTT CTG AGA TGA GTT TTT GTT CAG TAT AGG CCA TCT TGC TGC A 3′ was performed in presence of the RSV-BACL vector. In a second PCR the product was introduced into the pSec-Tag2 vector, fusing the BACL ectodomain to an N-terminal secretory targeting sequence and C-terminal c-myc- and hexahistidine-tags. The pSec-BACL vector was transfected into 293 cells using AppliFect (AppliChem) and stable cell clones were selected with 0.2 mg/ml hygromycin B. For large scale protein production 293 cells were cultured in a CELLine bioreactor (Integra, Fernwald, Germany). Soluble BACL-ectodomains were collected from the cell culture supernatant and purified by affinity chromatography with a Ni-NTA column (GE Healthcare, Munich, Germany). The purified protein was used for the immunization of chicken and subsequently, whole IgY antibodies were collected from the egg yolk (Gallus Immunotech, Fergus, Canada). BACL-specific IgY was further enriched by affinity purification with a BACL-loaded affinity column (Gallus Immunotech).

### Immunohistochemistry

Brain tissues from adult C57BL/6 mice were quick-frozen in liquid nitrogen and sagittal sections (thickness: 10 µm) were prepared on glass slides with a cryomicrotome (Leica). Single cells were stained as cytospin preparations on glass slides. Prior to staining tissues were fixed with acetone and bleached with the BLOXALL™ blocking solution (Vector Laboratories, Peterborough, UK). Tissues were treated with 3% (w/v) BSA to reduce non-specific binding and incubated with the primary antibody overnight at 4°C. For blocking experiments anti-BACL IgY was preincubated with soluble BACL 20 min on ice before addition to the slides. After staining, slides were washed in TBS and incubated with a biotinylated secondary antibody, followed by HRP-conjugated streptavidin (Dako, Hamburg, Germany) and detection with SIGMA*FAST*™ 3,3′-Diaminobenzidine tablets (Sigma). Slides were counterstained with Mayer's Hemalaun solution (AppliChem) and mounted in AquaTec (Merck, Darmstadt, Germany) for visualization.

### Flow Cytometry

Transfectants were incubated with either anti-FLAG antibody M2 (Sigma), anti-MICA antibody AMO1 [21], or mouse IgG1 isotype control at a final concentration of 5 µg/ml and secondary stainings were performed using PE-conjugated goat anti-mouse IgG antibody (Jackson ImmunoResearch Laboratories, Newmarket, UK). Anti-BACL or pre-immune IgY antibodies were used in a final concentration of 1.8 µg/ml and detected with FITC-conjugated donkey anti-chicken IgY antibodies (Gallus Immunotech). All samples were analysed with a FACSCanto™ II (BD Biosciences, Heidelberg, Germany).

### Immunoprecipitation and Immunoblotting

For immunoblot analysis, transiently transfected 293T cells were lysed using lysis buffer (50 mM Tris pH 8, 150 mM NaCl, 1% NP-40) containing the Complete protease inhibitor cocktail (Roche). Tissues from adult C57BL/6 mice or human brain tissue samples were passed through a 100 µm nylon mesh before lysis. For immunoprecipitation lysates were incubated with UltraLink Biosupport resin (Thermo Scientific, Rockford, USA) preloaded with anti-BACL or pre-immune IgY. Precipitation was carried out for 4 h at 4°C under gentle rotation. For deglycosylation, samples were treated with PNGase F (New England Biolabs, Frankfurt, Germany) according to the manufacturer’s instructions. 50 µg of total lysate or immunoprecipitates were separated by SDS-PAGE in a Mini-PROTEAN® Tetra Cell (Bio-Rad) and transferred to PVDF membranes (Roth, Arlesheim, Switzerland) by semi-dry blotting. Membranes were stained with anti-FLAG mAb M2 (Sigma) in a final concentration of 1 µg/ml followed by detection using a horseradish peroxidase (HRP)-conjugated goat anti-mouse IgG antibody (Jackson ImmunoResearch Laboratories). Precipitated BACL protein was detected by incubation with anti-BACL IgY in a final concentration of 1.8 µg/ml followed by incubation with HRP-conjugated donkey anti-chicken IgY antibody (Gallus Immunotech).

### Reporter Cell Assays

BWZ.36 reporter cells (1×10^5^ per well) were cultured overnight at 37°C either in Nunclon MaxiSorp™ 96-well plates (Thermo Scientific) precoated with anti-FLAG M2 mAb or IgG1 isotype control (10 µg/ml), or with 2×10^5^ target cells in 96-well round-bottom plates (Greiner Bio-one, Frickenhausen, Germany). Cells were lysed in presence of chlorophenol red-β-D-galactopyranoside (final concentration: 150 µM; Sigma) and β-galactosidase activity was determined by measuring absorbance at 595 nm. For positive control, BWZ.36 reporter cells were stimulated overnight with 10 ng/ml phorbol 12-myristate 13-acetate (PMA) and 1 µM ionomycin.

### Tumor Growth Experiments

C57BL/6J mice were purchased from Harlan Laboratories (The Netherlands) and housed in the Zentrale Forschungseinrichtung of the University of Frankfurt. 3x10^5^ cells of RMA-mock, RMA-BACL and RMA-MICA*07 cells [21] were injected s.c. into the left flank of the mice and tumor growth was monitored by measuring tumor surface with a metric caliper. Animals were sacrificed when tumors reached a size of ∼200 mm^2^ or on day 35 after tumor inoculation. Statistical analysis was performed by two-way ANOVA with a Bonferroni post-test using Prism 5 software (GraphPad, La Jolla, USA).

## Results

### The Orphan Gene *CLEC2L* Encodes for an Atypical and Highly Conserved C-type Lectin-like Receptor of the CLEC2 Family

**Figure 1:**
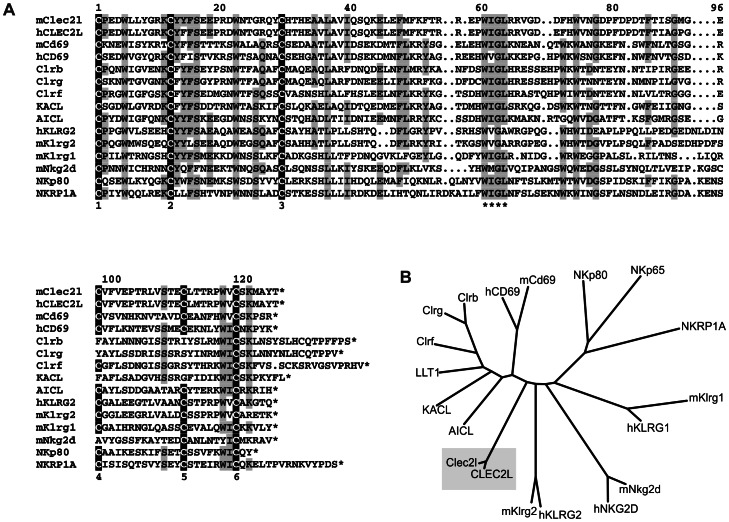
The *CLEC2L*-encoded CTLR is highly conserved and a member of the CLEC2 subfamily of CTLR. (A) Amino acid sequence alignment of the CTLDs of mouse Clec2l (mClec2l) and human CLEC2L (hCLEC2L) with NKC-encoded CLEC2 family members and other NKC-encoded CTLRs of human or mouse origin. Alignment starts with the first out of six highly conserved cysteines (Cys1 to Cys6) of the CTLD that are highlighted in black and numbered (bottom line) accordingly. The ‘WIGL’ motif of the hydrophobic core is indicated by asterisks (bottom line) and shaded, as are other conserved CTLD residues. Dots indicate sequence gaps. (B) Phylogenetic tree of the CTLD sequences shown in (A). Tree was generated with the PHYLIP program (http://evolution.genetics.washington.edu/phylip.html).

**Figure 2:**
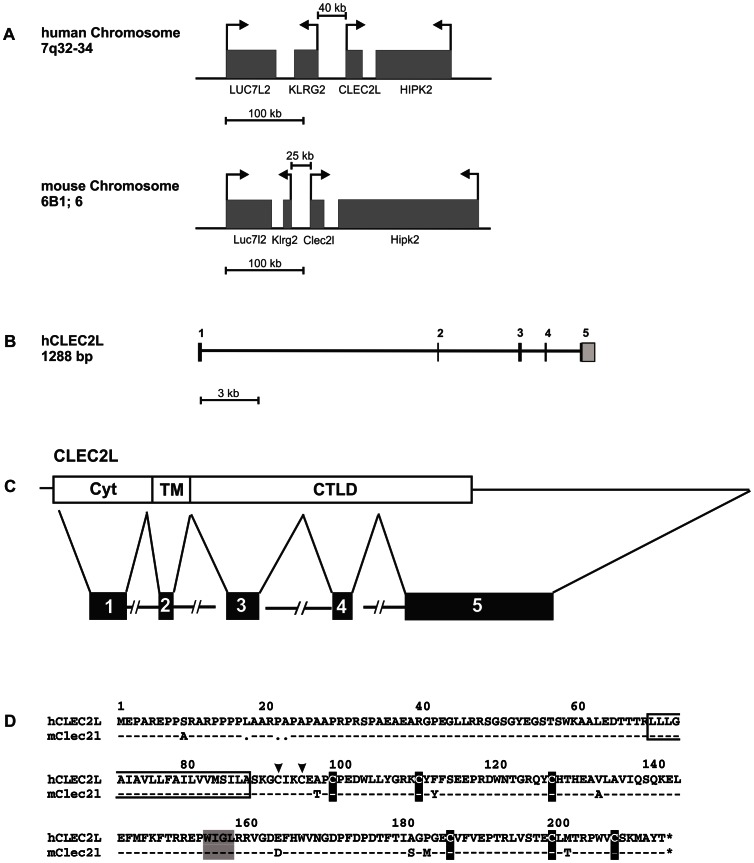
The *CLEC2L* gene and its products. (A) Map of the genomic region containing the *CLEC2L* gene in human and mouse. Boxes represent genes, arrows transcriptional orientation. The distance of the orphan genes *CLEC2L* and *KLRG2* is indicated. Adjacent genes are HIPK2 (homeodomain interacting protein kinase 2) and LUC7L2 (LUC7-like 2). (B) Schematic representation (true to scale) of the exon/intron structure of the human *CLEC2L* gene with the five exons numbered and the 3′ UTR sequence in light gray. (C) Schematic representation of protein domains encoded by the five exons. Cyt = cytoplasmic domain, TM = transmembrane domain, CTLD = C-type lectin-like domain. (D) Alignment of the human and mouse CLEC2L amino acid sequences. Dashes indicate identical amino acids, dots represent sequence gaps. The conserved cysteines of the CTLD are in black, the cysteines of the stalk are marked by arrows. The ‘WIGL’ motif is shaded and the predicted transmembrane region boxed.

Known members of the CLEC2 family are C-type lectin-like receptors (CTLRs) encoded in the Natural Killer Gene complex (NKC) which is located on human chromosome 12p13 and mouse chromosome 6F3. CLEC2 proteins include CD69, human AICL, KACL, and LLT1, and mouse Clr-molecules. Apart from CD69 no other CLEC2 family member has been described to be conserved in both species. Here we provide the first characterization of the CLEC2 gene *CLEC2L* that is highly conserved in man and mouse. The computational translation of the *CLEC2L* open reading frame revealed a type II transmembrane protein with a prototypical C-type lectin-like domain (CTLD) at the carboxyterminus. The latter is characterized by hallmark residues such as a central hydrophobic WIGL motif and six conserved cysteines that form three intramolecular disulfide bonds stabilizing the C-type lectin fold [2], [3] (**Figure 1A**). The CTLD of human CLEC2L exhibits highest sequence similarity to CD69 (40% identity/59% similarity), followed by the CTLD of LLT1 (35%/58%) and KACL (34%/55%), respectively. Amino acid alignments also revealed that CLEC2L uniquely shares with other CLEC2 family members a shortened sequence stretch between Gly84 and Cys88 (positions refer to CTLD of hCLEC2L; **Figure 1A**). Moreover, this analysis also revealed a strong conservation (95% identity) of the CTLDs of human and mouse CLEC2L proteins that by far exceeds the conservation of NKC-encoded CTLRs (**Figure 1B**). Interestingly, the *CLEC2L* gene is not located within the NKC but on human chromosome 7q34 and mouse chromosome 6B1, respectively, spanning ∼20 kb (**Figure 2A, B**). Further, a *CLEC2L* gene is documented for many other mammalian species including rodents, primates, even-toed ungulates, and carnivores (http://www.ncbi.nlm.nih.gov/gene/?term=clec2l). The human *CLEC2L* gene consists of five exons, with exon 1 encoding the cytoplasmic domain, exon 2 the transmembrane domain and exons 3, 4, and exon 5 the extracellular ectodomain giving rise to a protein of 214 amino acids (aa) with a predicted molecular mass of 23.9 kDa (**Figure 2C, D**). Amino acid sequences of the predicted mouse and human CLEC2L proteins only slightly differ at a total of 11 positions (**Figure 2D**). The extended CLEC2L cytoplasmic domain comprises about 70 aa and contains no known tyrosine-based signaling motifs but instead extended proline-rich regions and several serine residues that may relay extracellular signals. The transmembrane domain contains no charged amino acids arguing against association with signaling adaptors and is followed by a short stalk region (10 aa) with two cysteines involved in homodimerization (see below). Contrarily to other known CTLRs, the CLEC2L ectodomain contains no obvious sites prone to N- or O-linked glycosylation.

### *CLEC2L* Gives Rise to a Disulfide-linked Homodimeric Cell Surface CTLR

**Figure 3:**
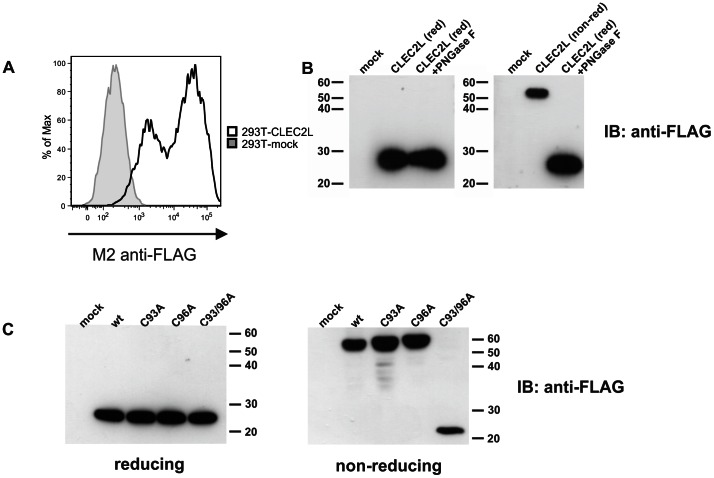
*CLEC2L* encodes a disulfide-linked homodimeric CTLR readily expressed on the cell surface. (A) 293T cells transiently transfected with carboxyterminally FLAG-tagged mouse CLEC2L cDNA (solid line) or vector control (filled) were analysed by flow cytometry using anti-FLAG mAb M2. (B, C) Immunoblots of 293T cells transiently transfected with FLAG-tagged mouse CLEC2L cDNA. CLEC2L proteins in cellular lysates were detected with mAb M2. Lysates of mock-transfected 293T cells (mock) served as controls. (B) Lysates of 293T cells expressing mouse CLEC2L proteins were separated by reducing (red; left panel) or non-reducing SDS-PAGE (non-red; right panel). Lysates were treated with PNGase F for protein deglycosylation (reducing conditions) where indicated. (C) Lysates of 293T cells expressing wildtype mouse CLEC2L (wt) or mouse CLEC2L mutants were separated by reducing (left panel) or non-reducing SDS-PAGE (right panel). Stalk region of CLEC2L was mutated, with cysteines 93 and/or 96 substituted by alanines.

To assess whether *CLEC2L,* like other CLEC2 genes, encodes a transmembrane C-type lectin-like receptor expressed at the cell surface, the *CLEC2L* open reading frame was cloned from brain cDNA of C57BL/6 mice and expressed in 293T cells. Flow cytometric analyses of transiently transfected 293T cells revealed that the *CLEC2L*-encoded FLAG-tagged CTLR is readily expressed at the cell surface (**Figure 3A**). Immunoblots of the corresponding 293T lysates revealed a protein with a molecular mass of ∼25 kDa matching the predicted molecular mass for the FLAG-tagged non-glycosylated CTLR. As expected, deglycosylation did not alter the apparent molecular mass (**Figure 3B**). Under non-reducing conditions, tagged CLEC2L proteins appeared with a molecular mass of ∼55 kDa in immunoblots (**Figure 3B**) suggesting the occurrence as disulfide-linked homodimers. The ectodomain of CLEC2L contains a total of eight cysteines with six of them engaged in the intramolecular stabilization of the conserved lectin-like fold (see above, **Figure 1**). To address whether the two cysteines of the stalk region (**Figure 2D**) account for CLEC2L dimerization, CLEC2L mutants with the respective alanine substitutions were generated. While both single mutants (C93A; C96A) migrated in non-reducing SDS-PAGE like wild-type CLEC2L, the double mutant (C93A/C96A) migrated like monomeric CLEC2L (**Figure 3C**), demonstrating that formation of stable CLEC2L homodimers is due to intermolecular disulfide bonds involving both cysteines of the CLEC2L stalk.

### Brain-associated Expression of *CLEC2L*

**Figure 4:**
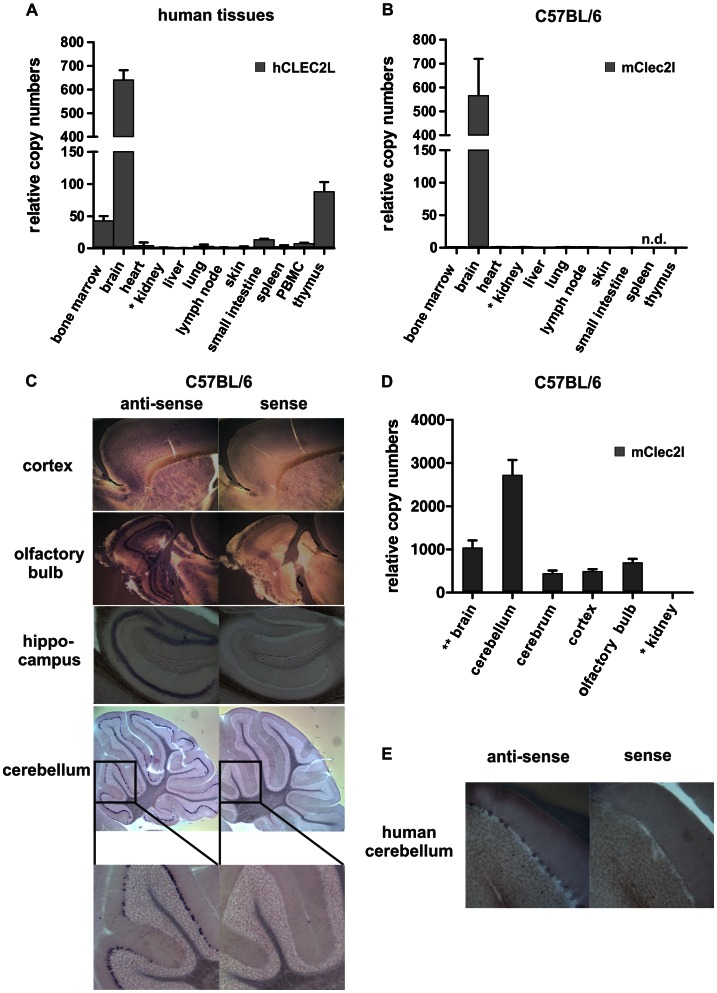
CLEC2L is predominantly expressed in the brain. (A) Quantitative RT-PCR of CLEC2L transcripts in various human tissues. Data are normalized to human TBP and arbitrarily set relative to CLEC2L transcript levels of kidney (*). (B) Quantitative RT-PCR of CLEC2L transcripts in various tissues of C57BL/6 mice. Data are normalized to 18S rRNA and arbitrarily set relative to CLEC2L transcript levels of kidney (*). (C) *In situ* hybridization of C57BL/6 mouse brain sections confirmed pronounced CLEC2L expression in the brain with particularly high levels in cerebellar Purkinje cells. *In situ* hybridization was performed using DIG-labeled sense (control) and anti-sense mouse CLEC2L probes. (D) Quantitative RT-PCR of CLEC2L transcripts in various brain regions isolated from C57BL/6 mice. Whole brain sample (**) was included for comparison. Data are normalized to 18S rRNA and arbitrarily set relative to CLEC2L transcript levels of kidney (*). (E) *In situ* hybridization of tissue sections of human cerebellum using DIG-labeled sense (control) and anti-sense human CLEC2L probes.

To obtain first insights into the functional context of CLEC2L we investigated CLEC2L tissue expression. Quantitative RT-PCR (qPCR) employing oligonucleotides amplifying parts of exon 4 and 5 of CLEC2L was used to address the abundance of CLEC2L transcripts in human and mouse tissues. Among the 24 analyzed human tissues, unexpectedly, high abundance of CLEC2L transcripts was only found for human brain (**Figure 4A**). Low levels of CLEC2L transcripts were detected in primary hematopoietic organs (bone marrow and thymus) while other tissues only contained very low or undetectable levels of CLEC2L transcripts. To assess evolutionary conservation of this tissue-restricted expression pattern, various tissues of C57BL/6 mice were examined for CLEC2L transcripts. Again, the most abundant CLEC2L expression was found for mouse brain, while all other investigated tissues contained low or undetectable levels of CLEC2L transcripts (**Figure 4B**). Similar results were obtained in analyses of tissues from BALB/c mice (data not shown). The abundant CLEC2L expression in the brain prompted further studies to assign the expression to a given cell type. Thus, *in situ* hybridization was employed to visualize CLEC2L transcripts in brain sections of adult C57BL/6 mice. Using a probe covering the entire CLEC2L coding sequence, CLEC2L expression was clearly detectable in the cortex, the olfactory bulb and the hippocampus and showed highest levels for cerebellar Purkinje cells (**Figure 4C**). This expression pattern was confirmed by hybridization with a non-overlapping probe derived from 3′ UTR of CLEC2L (data not shown) and further corroborated by qPCR of mouse brain compartments (**Figure 4D**). Likewise, *in situ* hybridization of sections of human cerebellum revealed CLEC2L expression by the Purkinje cell layer (**Figure 4E**). Collectively, these data demonstrate predominant CLEC2L expression in the brain, with marginal or no expression in other tissues. Hence, the CLEC2L-encoded CTLR was termed BACL (brain-associated C-type lectin).

### Neuronal BACL Expression

**Figure 5:**
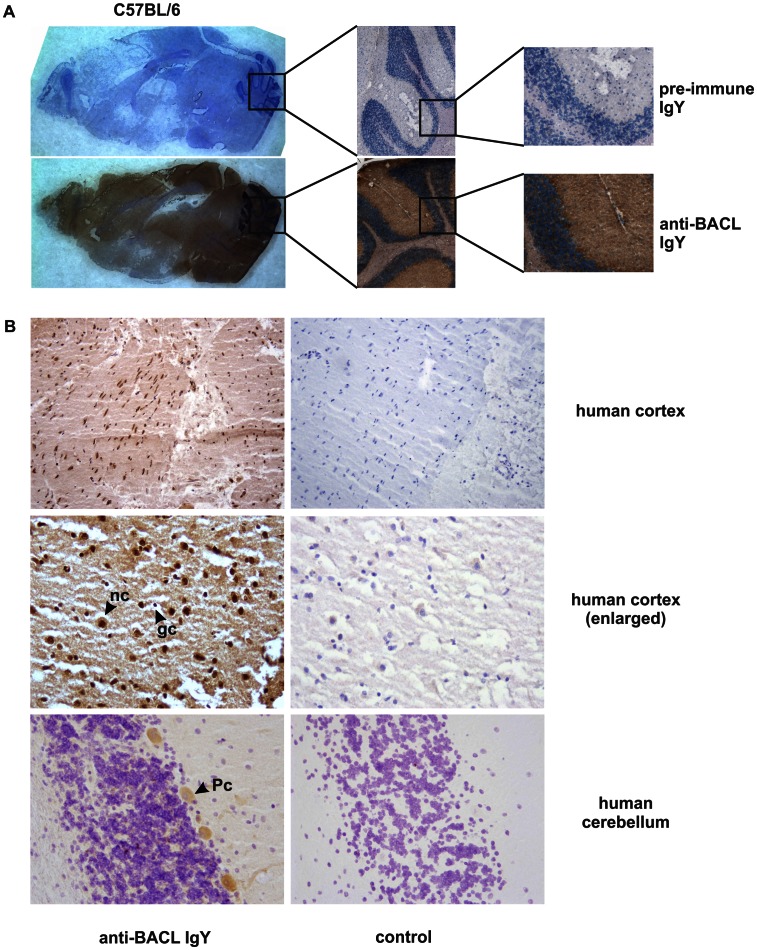
Broad brain presence of BACL is associated with neuronal tissue. (A) BACL proteins in mouse brain. Cryosections of whole mouse brain (C57BL/6 mice) were either stained with BACL-specific or control (pre-immune) chicken IgY followed by biotin-conjugated anti-chicken IgY antibodies. Enlarged sections of mouse cerebellum highlight BACL expression (brown) by Purkinje cells. Nuclei are counterstained (blue). (B) BACL proteins in human brain. Cryosections of human cortex and cerebellum were stained with BACL-specific IgY followed by biotin-conjugated anti-chicken IgY antibodies or with secondary antibody only (control). Apparent BACL expression (brown) by cortical neurons and Purkinje cells. Nuclei are counterstained (blue) (nc = neuronal cell, gc = glial cell, Pc = Purkinje cell).

**Figure 6:**
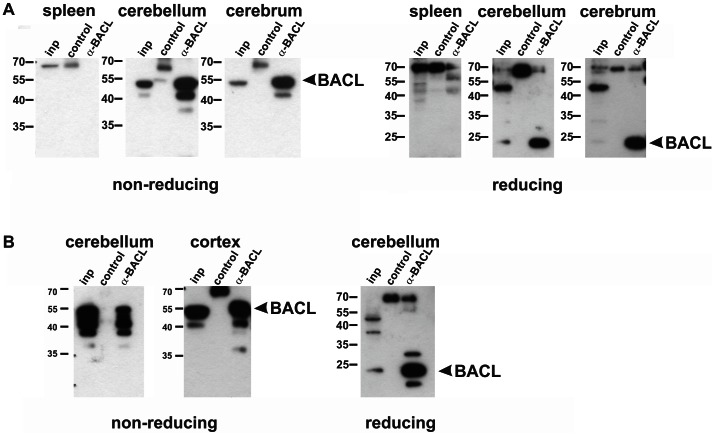
Brain-associated BACL molecules. (A, B) Depiction of BACL proteins from mouse and human brain. (A) BACL proteins immunoprecipitated from cellular lysates of mouse cerebrum and cerebellum, respectively, were subjected to reducing (right) or non-reducing SDS-PAGE (left) and detected by immunoblotting with anti-BACL IgY. No BACL was detected in lysates of spleens or in control immunoprecipitates (pre-immune IgY). (B) BACL proteins immunoprecipitated from cellular lysates of human cerebellum and cortex, respectively, were subjected to reducing (right) or non-reducing SDS-PAGE (left) and detected by immunoblotting with anti-BACL IgY. No BACL was detected in control immunoprecipitates (pre-immune IgY). (A, B) Cellular lysates were included as input controls (inp).

For detection of endogenous BACL expression there was a need for a specific reagent as no commercial antibodies were available. To this aim, soluble human BACL ectodomains were expressed in stably transfected 293 cells, purified from supernatants by affinity chromatography (**Figure S1A**), and subsequently used for immunization of chicken. Resulting affinity-purified chicken IgY was shown to specifically detect ectopically expressed both human and mouse BACL in flow cytometry, immunoblotting, and immunocytochemistry, respectively (**Figure S1B–D**). Hence, BACL-specific IgY was employed for immunohistochemical analyses of mouse brain. As shown in **Figure 5A**, BACL-specific IgY, but not control IgY, clearly stained Purkinje cells and other neuron-rich brain regions. This staining could be blocked by pre-incubation with soluble BACL (data not shown). In conjunction with the results of the *in situ* hybridization (**Figure 4C**) these data clearly demonstrate prominent BACL expression in the mouse brain. Similarly, immunohistochemical analyses of human brain regions revealed BACL expression by cortical neurons and cerebellar Purkinje cells (**Figure 5B**). Using anti-BACL IgY, BACL proteins were immunoprecipitated from lysates of brains of man and mice (**Figure 6**) and shown to display molecular masses upon reducing (∼24 kDa) or non-reducing SDS-PAGE (∼50 kDa) corresponding to ectopically expressed BACL (**Figure 3**). BACL precipitation was further confirmed by mass spectrometry analysis of the precipitate (data not shown). These results demonstrate that expression of BACL in the brain can be attributed to neurons raising the question of the functional significance thereof.

### Genetic Linkage of BACL with the Orphan CTLR KLRG2

**Figure 7:**
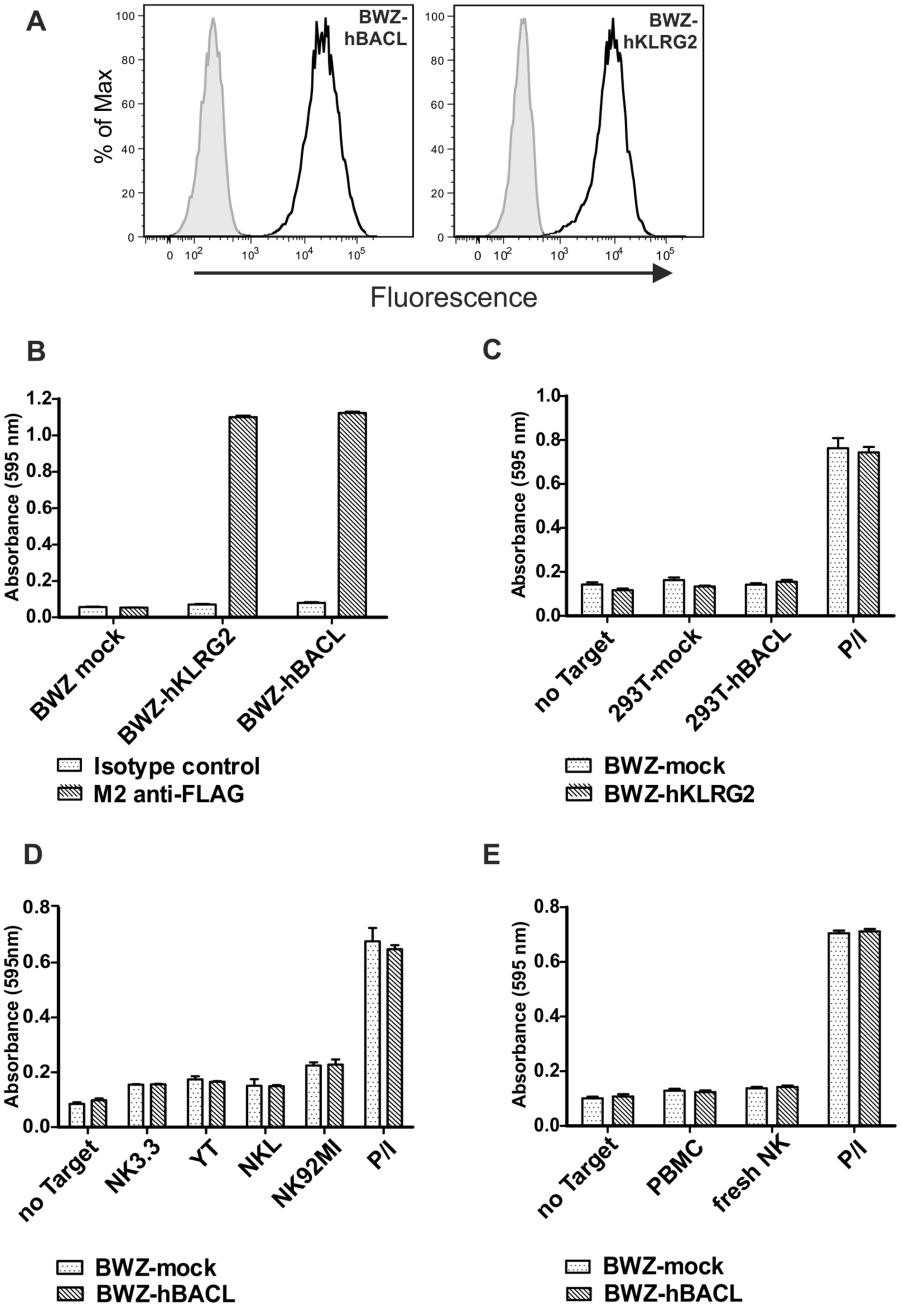
Reporter assays failed to provide evidence for a BACL receptor. (A) Bright expression of FLAG-tagged hBACL-CD3ζ or hKLRG2-CD3ζ chimera on stably transfected BWZ.36 cells detected by flow cytometry using anti-FLAG mAb M2 (solid lines). IgG1 isotype control stainings are filled. (B) Functional responsiveness of BACL-CD3ζ or KLRG2-CD3ζ expressing BWZ.36 reporter cells upon overnight stimulation with immobilized mAb M2, but not with control IgG1. (C) BWZ.36-hKLRG2-CD3ζ cells or mock-transfected BWZ.36 controls were cultured overnight with 293T cells transiently transfected with hBACL or mock-transfected 293T cells. (D, E) Mock-transfected BWZ.36 controls or BWZ.36-hBACL-CD3ζ reporter cells were cultured overnight with indicated NK cell lines (D) or freshly isolated human PBMC or NK cells (E). (C – E) Treatment of BWZ.36 cells with ionomycin and PMA (P/I) served as a positive control.

Genetic linkage of certain C-type lectin-like receptor-ligand pairs in the mouse NKC was first reported in 2003 by Yokoyama and colleagues [13]. Since then, a couple of other interactions between genetically linked CTLRs of the NKRP1 and CLEC2 families has been described for the murine NKC, but also for the human NKC [9], [10], [14]–[18]. As pointed out above, in close proximity to the *CLEC2L* locus in both man and mouse there is a locus of an uncharacterized orphan CTLR termed *KLRG2*. Although this designation suggest a close relationship to the CTLR KLRG1, the putative CTLR KLRG2 is not more related to KLRG1 than to NKG2D (**Figure 1B**). However, due to the genetic linkage, we hypothesized that KLRG2 may represent a receptor for BACL. To directly test this possibility, BWZ.36 reporter cells were generated expressing FLAG-tagged fusion proteins comprising the ectodomains of BACL and KLRG2, respectively, merged with cytoplasmic domains of CD3ζ for signaling. Both, BWZ.36-BACL and BWZ.36-KLRG2 reporter cells expressed the respective fusion proteins at high levels at the cell surface as determined by flow cytometry (**Figure 7A**) and both chimeric receptors strongly stimulated BWZ.36 cells upon antibody-mediated crosslinking (**Figure 7B**). However, no activity was detected when 293T cells expressing the human BACL protein were co-cultured with BWZ.36-KLRG2 reporter cells (**Figure 7C**). Hence, BACL and KLRG2 did not interact, at least not in the context of this reporter assay. However, it cannot be ruled out that certain posttranslational modifications or other modifiers absent in BWZ.36 or 293T cells may be a prerequisite of functional KLRG2-BACL interaction. We further investigated a possible engagement of BACL by receptors present on NK cells. As shown in **Figure 7D and 7E**, none of the co-cultures of BWZ.36-BACL with any of the indicated NK cell lines or freshly isolated NK cells or PBMC resulted in a significant response by BWZ.36-BACL reporter cells.

### BACL does not Affect Tumor Growth

**Figure 8:**
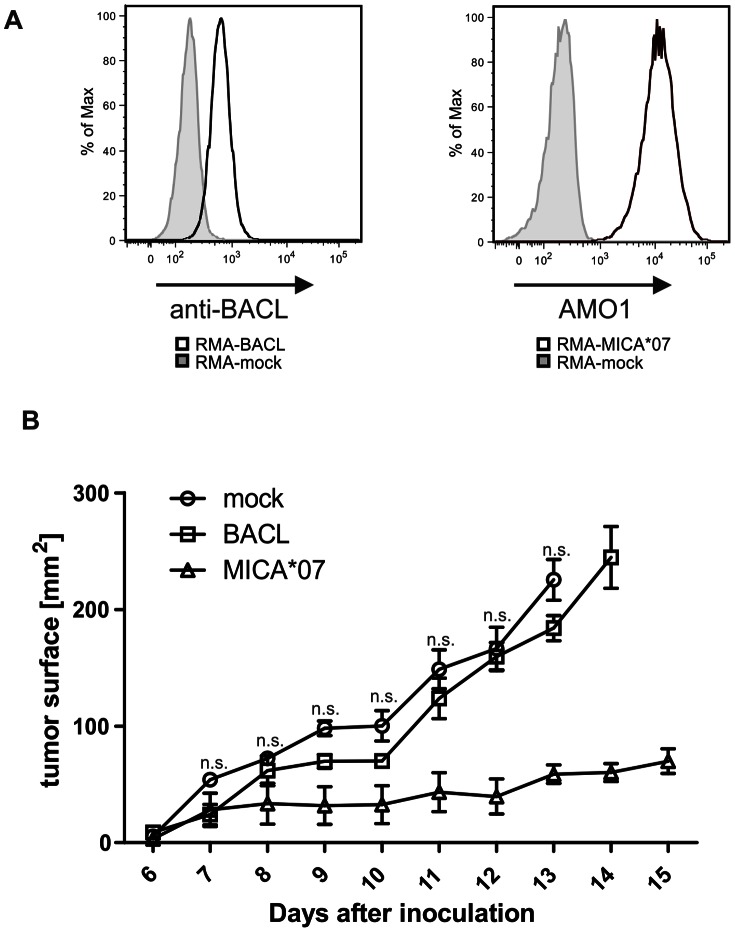
Tumor-associated BACL expression does not affect tumor growth *in vivo*. (A) Ectopic expression of BACL or MICA by the respective RMA transfectants as revealed by flow cytometry with anti-BACL IgY or anti-MICA mAb AMO1 (solid lines). Stainings of mock-transfected RMA for control (filled). (B) 3x10^5^ RMA-BACL, RMA-MICA*07, or RMA-mock cells were subcutaneously inoculated in syngeneic C57BL/6 mice and tumor growth was monitored for the indicated period with a metric caliper. While differences in tumor size were significant (starting from day 9) between RMA-mock (n  = 4) and RMA-MICA*07 (positive control, n  = 3), there was no significant difference (n.s.) in tumor size between RMA-mock and RMA-BACL (n  = 4). Error bars represent SEM.

NK cells are known to play a significant role in tumor surveillance. Extensive work has been done to study NKG2D-mediated tumor rejection *in vivo* exploiting syngeneic tumor models. Overexpression of mouse and human NKG2D ligands such as Rae-1, H60, MULT-1, and MICA on tumor cells resulted in a retarded tumor growth or tumor rejection due to NK-cell mediated cytolysis [21]–[23]. This prompted us to test whether BACL expression may evoke immune responses altering tumor cell growth *in vivo*. To this aim, we monitored subcutaneous tumor growth of BACL-overexpressing RMA cells (**Figure 8A**) in a syngeneic mouse tumor model. Whereas growth of MICA-expressing RMA cells was significantly retarded as expected, growth of BACL-expressing RMA was comparable to mock-transfected cells (**Figure 8B**). Similar results were obtained with varying numbers of RMA tumor cells inoculated (data not shown). These results did not reveal any significant immune response provoked by BACL expression, at least in this tumor rejection model.

## Discussion

Here we provide the first description of the *CLEC2L* gene and its product, a homodimeric C-type lectin-like cell surface receptor that was termed BACL due to its brain-associated expression. Sequence alignments define BACL as a member of the CLEC2 family of C-type lectin-like receptors that also includes CD69, human LLT1 (*CLEC2D*), AICL (*CLEC2B*) and KACL (*CLEC2A*), as well as mouse Clr molecules. Both sequence homology and certain hallmarks of the CTLD of BACL allow for this classification. However, BACL uniquely differs from other members of the CLEC2 family by several criteria: (i) Contrary to most CLEC2 family members BACL is highly conserved among mammals, (ii) BACL is not encoded in the NKC, (iii) BACL is devoid of N-glycosylation, (iv) BACL expression predominates in the brain where most other CLEC2 family members are not or just barely expressed [12].

Such tissue-restricted expression is reminiscent of several other CLEC2 family members such as KACL, which is almost exclusively expressed by human keratinocytes [10], or Clr-d and Clr-f which are specifically expressed in the eye and intestine of mice, respectively [12]. Notably, none of the numerous Clr molecules has been found to be substantially expressed in the mouse brain [12] underlining the peculiarity of BACL among the CLEC2 family members.

Combining *in situ* hybridization and immunohistochemistry, BACL expression was attributed to neurons, with high levels of BACL mRNA in Purkinje cells, while no BACL mRNA was detected in the granular layer of the cerebellum. The expression in the cerebellum was higher than in the cerebrum, likely due to the exceptionally high levels of BACL transcripts in Purkinje cells that represent only less than 0.1% of the total cellular content of the cerebellum [24]. Purkinje cells, GABAergic projection neurons in the cerebellar cortex, are central components of the cerebellar circuitry and essential for motor control and coordination as well as for specific forms of motor learning [25], [26]. The significance of the strong expression of BACL associated with Purkinje cells remains to be determined.

BACL may be involved in the cellular cross-talk of Purkinje cells and other neurons with brain-resident cells or brain-infiltrating immune cells involving the BACL CTLD for interaction. Considering that NKC-encoded CLEC2 family members have been shown to interact with genetically linked CTLR of the NKRP1 family, we tested the possibility that the tightly linked orphan CTLR KLRG2 may represent a ligand of BACL. However, reporter assays did not reveal a physical interaction of BACL with KLRG2. Having in mind the immunological function of other CLEC2 family members, we also considered an immune-related function for BACL. But neither *in vitro* reporter cell assays suggested the presence of a BACL ligand on peripheral blood immune cells nor inoculation of BACL-expressing tumor cell lines provided *in vivo* evidence for BACL-mediated immune recognition. However, obviously, an immunological function remains possible and of interest, as neurons are specifically targeted by certain viruses, some of which persist for lifetime [27]. On the other hand, there is emerging evidence that immune-related molecules expressed in the brain may serve functions other than immune responses. For example, molecules structurally related to cytokines, complement factors, and MHC class I molecules described in developing and adult brain were linked to development and synaptic plasticity [28]–[31]. Most evidence for the brain-associated function of MHC class I-related molecules and their receptors was gained from studies of knock-out mice (B2m/TAP1^−/−^, Kb/Db^−/−^, CD3ζ^−/−^, PirB^−/−^) [32].

Identification of a ligand of BACL will be of major importance to delineate the functional relevance of BACL. Interaction of BACL with a putative ligand also may transmit signals into neurons as the extended and conserved cytoplasmic domain of BACL contains long stretches of proline-rich motifs that may recruit SH3-domain-containing proteins. Obviously, generation of *CLEC2L* knock-out mice likely will further our understanding of the function of this novel CTLR.

### Concluding Remarks

The highly conserved sequence and tissue expression of the newly described, unusual CLEC2 family member BACL argues in favour of a functional significance in the context of neurons, but whether this function is associated with immune recognition or rather with development and plasticity of neuronal networks remains to be determined by future studies.

## Supporting Information

Figure S1 **Generic detection of BACL proteins by BACL-specific IgY.** (A) Soluble BACL ectodomains purified by affinity chromatography were subjected to reducing (right) and non-reducing SDS-PAGE (left) and visualized by InstantBlue staining. (B) Polyclonal anti-BACL chicken IgY was used to detect purified soluble BACL ectodomains (sBACL) or mouse (mBACL) and human BACL (hBACL) proteins in lysates of transfected 293 cells after reducing SDS-PAGE. (C) BACL-specific IgY specifically binds to human BACL (left) and mouse BACL (right) ectopically expressed on BWZ.36 cells or 293 cells, respectively (upper panels). Stainings with pre-immune IgY or of mock-transfected cells are shown as negative controls. Corresponding stainings of the FLAG-tagged BACL proteins with mAb M2 (or isotype control) are shown for comparison (lower panels). (D) BACL-specific IgY detects ectopically expressed hBACL or mBACL also on cytospins. Pre-immune IgY stainings are shown for control.
(TIF)

## References

[1] Yokoyama WM, Seaman WE (1993) The Ly-49 and NKR-P1 gene families encoding lectin-like receptors on natural killer cells: the NK gene complex. Annu Rev Immunol 11: 613–635. 8476574 10.1146/annurev.iy.11.040193.003145

[2] Yokoyama WM, Plougastel BF (2003) Immune functions encoded by the natural killer gene complex. Nat Rev Immunol 3: 304–316. 12669021 10.1038/nri1055

[3] Zelensky AN, Gready JE (2005) The C-type lectin-like domain superfamily. FEBS J 272: 6179–6217. 16336259 10.1111/j.1742-4658.2005.05031.x

[4] Vogler I, Steinle A (2011) Vis-a-vis in the NKC: genetically linked natural killer cell receptor/ligand pairs in the natural killer gene complex (NKC). J Innate Immun 3: 227–235. 21422751 10.1159/000324112

[5] Lanier LL, Chang C, Phillips JH (1994) Human NKR-P1A. A disulfide-linked homodimer of the C-type lectin superfamily expressed by a subset of NK and T lymphocytes. J Immunol 153: 2417–2428. 8077657

[6] Aust JG, Gays F, Mickiewicz KM, Buchanan E, Brooks CG (2009) The expression and function of the NKRP1 receptor family in C57BL/6 mice. J Immunol 183: 106–116. 19535641 10.4049/jimmunol.0804281

[7] Vitale M, Falco M, Castriconi R, Parolini S, Zambello R, et al (2001) Identification of NKp80, a novel triggering molecule expressed by human NK cells. Eur J Immunol 31: 233–242. 11265639 10.1002/1521-4141(200101)31:1<233::AID-IMMU233>3.0.CO;2-4

[8] Kuttruff S, Koch S, Kelp A, Pawelec G, Rammensee HG, et al (2009) NKp80 defines and stimulates a reactive subset of CD8 T cells. Blood 113: 358–369. 18922855 10.1182/blood-2008-03-145615

[9] Welte S, Kuttruff S, Waldhauer I, Steinle A (2006) Mutual activation of natural killer cells and monocytes mediated by NKp80-AICL interaction. Nat Immunol 7: 1334–1342. 17057721 10.1038/ni1402

[10] Spreu J, Kuttruff S, Stejfova V, Dennehy KM, Schittek B, et al (2010) Interaction of C-type lectin-like receptors NKp65 and KACL facilitates dedicated immune recognition of human keratinocytes. Proc Natl Acad Sci U S A 107: 5100–5105. 20194751 10.1073/pnas.0913108107 PMC2841919

[11] Rosen DB, Cao W, Avery DT, Tangye SG, Liu YJ, et al (2008) Functional consequences of interactions between human NKR-P1A and its ligand LLT1 expressed on activated dendritic cells and B cells. J Immunol 180: 6508–6517. 18453569 10.4049/jimmunol.180.10.6508 PMC2577150

[12] Zhang Q, Rahim MM, Allan DS, Tu MM, Belanger S, et al (2012) Mouse Nkrp1-Clr gene cluster sequence and expression analyses reveal conservation of tissue-specific MHC-independent immunosurveillance. PLoS One 7: e50561. 23226525 10.1371/journal.pone.0050561 PMC3514311

[13] Iizuka K, Naidenko OV, Plougastel BF, Fremont DH, Yokoyama WM (2003) Genetically linked C-type lectin-related ligands for the NKRP1 family of natural killer cell receptors. Nat Immunol 4: 801–807. 12858173 10.1038/ni954

[14] Carlyle JR, Jamieson AM, Gasser S, Clingan CS, Arase H, et al (2004) Missing self-recognition of Ocil/Clr-b by inhibitory NKR-P1 natural killer cell receptors. Proc Natl Acad Sci U S A 101: 3527–3532. 14990792 10.1073/pnas.0308304101 PMC373496

[15] Kveberg L, Dai KZ, Inngjerdingen M, Brooks CG, Fossum S, et al (2011) Phylogenetic and functional conservation of the NKR-P1F and NKR-P1G receptors in rat and mouse. Immunogenetics 63: 429–436. 21409442 10.1007/s00251-011-0520-1 PMC3111725

[16] Chen P, Belanger S, Aguilar OA, Zhang Q, St-Laurent A, et al (2011) Analysis of the mouse 129-strain Nkrp1-Clr gene cluster reveals conservation of genomic organization and functional receptor-ligand interactions despite significant allelic polymorphism. Immunogenetics 63: 627–640. 21667046 10.1007/s00251-011-0542-8

[17] Rosen DB, Bettadapura J, Alsharifi M, Mathew PA, Warren HS, et al (2005) Cutting edge: lectin-like transcript-1 is a ligand for the inhibitory human NKR-P1A receptor. J Immunol 175: 7796–7799. 16339513 10.4049/jimmunol.175.12.7796

[18] Aldemir H, Prod'homme V, Dumaurier MJ, Retiere C, Poupon G, et al (2005) Cutting edge: lectin-like transcript 1 is a ligand for the CD161 receptor. J Immunol 175: 7791–7795. 16339512 10.4049/jimmunol.175.12.7791

[19] Sanderson S, Shastri N (1994) LacZ inducible, antigen/MHC-specific T cell hybrids. Int Immunol. 6: 369–376. 10.1093/intimm/6.3.369 8186188

[20] Heine P, Dohle E, Bumsted-O'Brien K, Engelkamp D, Schulte D (2008) Evidence for an evolutionary conserved role of homothorax/Meis1/2 during vertebrate retina development. Development 135: 805–811. 18216174 10.1242/dev.012088

[21] Wiemann K, Mittrucker HW, Feger U, Welte SA, Yokoyama WM, et al (2005) Systemic NKG2D down-regulation impairs NK and CD8 T cell responses in vivo. J Immunol 175: 720–729. 16002667 10.4049/jimmunol.175.2.720

[22] Diefenbach A, Jensen ER, Jamieson AM, Raulet DH (2001) Rae1 and H60 ligands of the NKG2D receptor stimulate tumour immunity. Nature 413: 165–171. 11557981 10.1038/35093109 PMC3900321

[23] Cerwenka A, Baron JL, Lanier LL (2001) Ectopic expression of retinoic acid early inducible-1 gene (RAE-1) permits natural killer cell-mediated rejection of a MHC class I-bearing tumor in vivo. Proc Natl Acad Sci U S A 98: 11521–11526. 11562472 10.1073/pnas.201238598 PMC58762

[24] Hawkes R, Gravel C (1991) The modular cerebellum. Prog Neurobiol 36: 309–327. 1871318 10.1016/0301-0082(91)90004-k

[25] Voogd J, Glickstein M (1998) The anatomy of the cerebellum. Trends Neurosci 21: 370–375. 9735944 10.1016/s0166-2236(98)01318-6

[26] Hirata Y, Katagiri K, Tanaka Y (2012) Direct causality between single-Purkinje cell activities and motor learning revealed by a cerebellum-machine interface utilizing VOR adaptation paradigm. Cerebellum 11: 455–456. 22528967 10.1007/s12311-012-0385-3

[27] Kristensson K (2011) Microbes' roadmap to neurons. Nat Rev Neurosci 12: 345–357. 21587289 10.1038/nrn3029

[28] Stephan AH, Barres BA, Stevens B (2012) The complement system: an unexpected role in synaptic pruning during development and disease. Annu Rev Neurosci 35: 369–389. 22715882 10.1146/annurev-neuro-061010-113810

[29] Shatz CJ (2009) MHC class I: an unexpected role in neuronal plasticity. Neuron 64: 40–45. 19840547 10.1016/j.neuron.2009.09.044 PMC2773547

[30] Boulanger LM (2009) Immune proteins in brain development and synaptic plasticity. Neuron 64: 93–109. 19840552 10.1016/j.neuron.2009.09.001

[31] Garay PA, McAllister AK (2010) Novel roles for immune molecules in neural development: implications for neurodevelopmental disorders. Front Synaptic Neurosci 2: 136. 21423522 10.3389/fnsyn.2010.00136 PMC3059681

[32] Elmer BM, McAllister AK (2012) Major histocompatibility complex class I proteins in brain development and plasticity. Trends Neurosci 35: 660–670. 22939644 10.1016/j.tins.2012.08.001 PMC3493469

