# Supplementary figures and images for: BACL Is a Novel Brain-Associated, Non-NKC-Encoded Mammalian C-Type Lectin-Like Receptor of the CLEC2 Family
Source: PLoS One. 2013 Jun 11;8(6):e65345. doi: 10.1371/journal.pone.0065345 (PMC3679072; doi:10.1371/journal.pone.0065345)

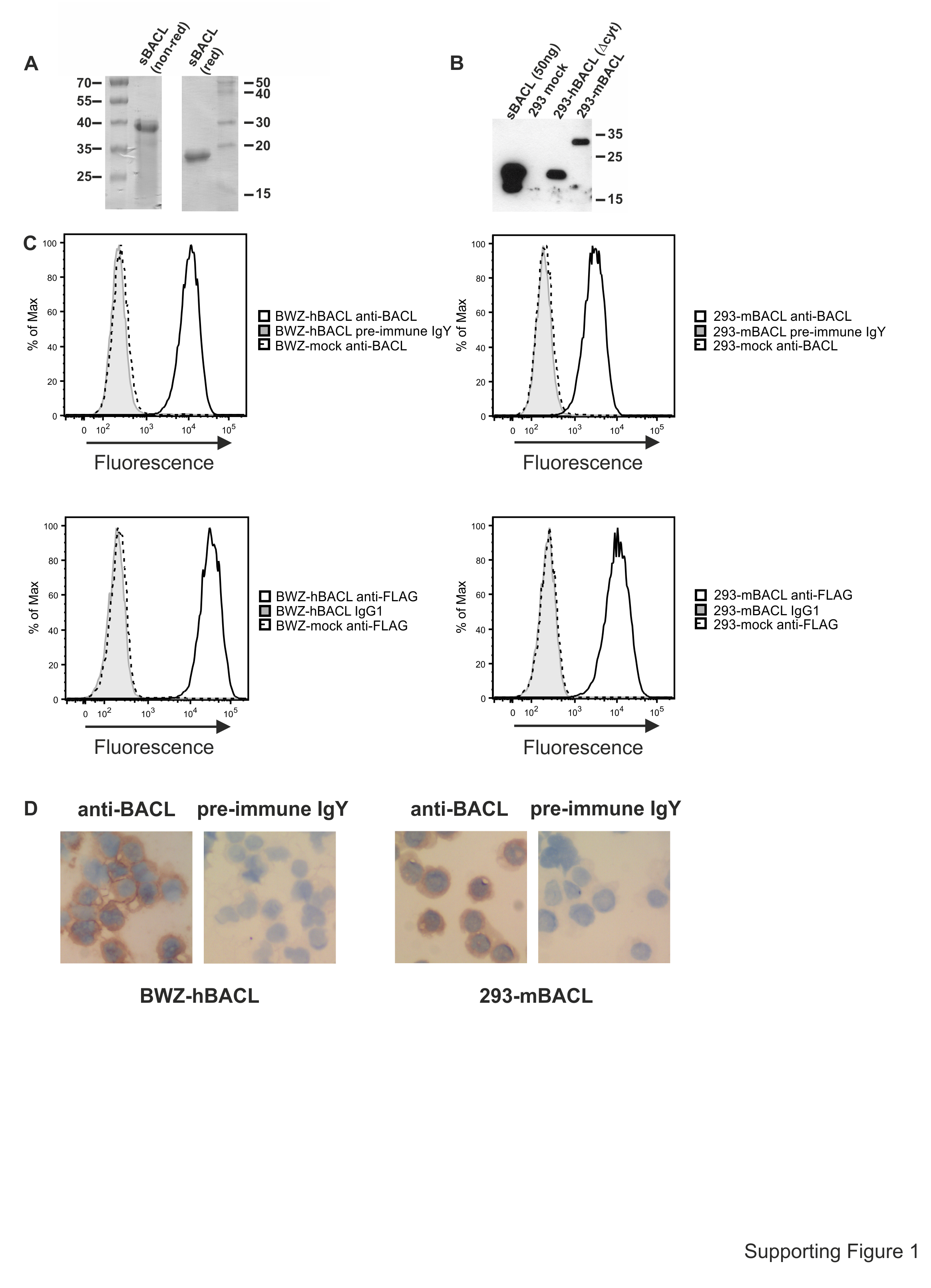

Supplement: Figure S1 — Generic detection of BACL proteins by BACL-specific IgY. (A) Soluble BACL ectodomains purified by affinity chromatography were subjected to reducing (right) and non-reducing SDS-PAGE (left) and visualized by InstantBlue staining. (B) Polyclonal anti-BACL chicken IgY was used to detect purified soluble BACL ectodomains (sBACL) or mouse (mBACL) and human BACL (hBACL) proteins in lysates of transfected 293 cells after reducing SDS-PAGE. (C) BACL-specific IgY specifically binds to human BACL (left) and mouse BACL (right) ectopically expressed on BWZ.36 cells or 293 cells, respectively (upper panels). Stainings with pre-immune IgY or of mock-transfected cells are shown as negative controls. Corresponding stainings of the FLAG-tagged BACL proteins with mAb M2 (or isotype control) are shown for comparison (lower panels). (D) BACL-specific IgY detects ectopically expressed hBACL or mBACL also on cytospins. Pre-immune IgY stainings are shown for control. (TIF) [file pone.0065345.s001.tif]
